# Harnessing artificial neural networks for accurate PV system parameters determination: radiation, temperature, and MPPT

**DOI:** 10.1038/s41598-026-40175-5

**Published:** 2026-03-23

**Authors:** Islam M. Abdelqawee, Mohamed Selmy, Mahmoud N. ALI, Alzhraa A. Abdelfattah, Wael Mamdouh

**Affiliations:** 1 Electrical Engineering Department, Faculty of Engineering at Shoubra, Benha University, Cairo, Egypt; 2 Egyptian Academy for Engineering and Advanced Technology, Cairo, Egypt

**Keywords:** ANN, MPPT, Photovoltaic, P&O, Incremental conductance, Energy science and technology, Engineering

## Abstract

Photovoltaic (PV) systems are increasingly significant in modern electrical energy applications. Extracting the maximum power from PV modules with high efficiency requires measuring temperature (T) and irradiance (G), which often demands sensors that increase the overall system cost. Furthermore, tracking the PV maximum power point (MPP) under varying T and G presents a considerable challenge. Conventional MPPT techniques require a long time to reach the MPP and can exhibit fluctuations during operation. To address these challenges, this work proposes a novel two-stage maximum power point tracking (MPPT) strategy. In the first stage, T and G are estimated using an artificial neural network (ANN) based on the measured PV open-circuit voltage and short-circuit current, thereby reducing system cost. The first proposed stage is compared with Newton Raphson and Open circuit voltage methods (VOC) in terms of T and G errors. In the second stage, the MPP is determined directly by ANN under varying T and G, minimizing tracking time and fluctuations. This stage is compared with Fuzzy logic control (FLC), Perturb and observe (P&O), Fixed increment conductance (FIC) and Variable increment conductance (VIC) in terms of efficiency, time capture (TC), and steady-state error. Simulation results demonstrate high tracking efficiency (99.99%), fast settling time (0.007 s), and low voltage/current ripples (0.018/0.12). Comparison with FLC (99.1%, 0.0275s), P&O (98.7%, 0.0322s), FIC (98.78%, 0.0517s), and VIC (98.81%, 0.0342s) confirms the best performance of the proposed method. The proposed ANN-based method is applied to simulate the system for three case studies. In the first case, predefined data are utilized, while in the second case, real T and G data from Hurghada, Egypt are employed. Third case is an experimental setup established to validate the performance of the proposed ANN strategy. The result of the proposed system was evaluated using MATLAB/Simulink.

## Introduction

The changes in atmospheric conditions, such as T and solar G throughout the day have a great impact on the PV array efficiency. Therefore, studying the effect of temperature and solar irradiance on PV panels is crucial. The measurements of T and G for the PV array are essential for effectively extracting MPP. In some circumstances, measurements are inaccurate. Therefore, alternative measurement methods should be used instead^1^. The usage of the pyranometer or reference solar cells is limited. This is owing to the pyranometer’s limitations due to its cost, installation, and maintenance difficulty in providing accuracy^2,3^. To better measure performance, it is occasionally required to use an extra number of sensors. Therefore, techniques for lowering sensors and costs as well as maintaining sufficient solar radiation detection are needed efficiently^4^.

In^2^, a method for estimating solar irradiance in W/m^2^ was provided. This method was based on the short circuit current that the PV module produces. In^4^, mathematical methods were used to calculate sun irradiance using the PV model. As a result, a relationship between solar irradiation and the PV module’s output voltage was established; however, because the estimation relied on an accurate PV model and parameters that change with temperature and aging, its accuracy decreases under dynamic environmental conditions.

In^5^, the conventional method such as Newton-Raphson was used to estimate solar radiation. The Thévenin equivalent circuit for PV modules has several limitations: it is a linear or quasi-linear approximation that cannot fully capture the nonlinear behavior of PV cells, leading to reduced accuracy under changing irradiance, temperature. It is also difficult to derive accurate I–V characteristics and is valid only within a limited linear range, making it unsuitable for precise real-time solar irradiance estimation.

In^2^, the conventional open circuit voltage (VOC) method was used to estimate The Open-Circuit Voltage method, while theoretically reliable, faced practical limitations: it was difficult to apply in real-world conditions, its accuracy depended on precise knowledge of key parameters such as Voc, its temperature coefficient, and the diode quality factor, and achieving high accuracy (uncertainty ≤ 1 °C) required controlled laboratory measurements, making it less suitable for real-time applications^6^. This literature is provided for the first stage.

The following literature is included to support the second step of the proposed method. Due to the continuous change in the environmental condition, primarily T and G, the P-V characteristics curve shows a non-linear maximum power point (MPP). To guarantee that the maximum power is always extracted, the MPPT is employed in conjunction with the power converter. Exceptional forms of MPPT strategies were advanced and employed. These techniques may be differentiated depending on the used sensors, convergence speed, cost, variety of effectiveness, implementation experimental necessities, and popularity.

These techniques are categorized into two categories: one is classical, which consists of direct and indirect strategies. The second is modern, which is financed by Artificial intelligence techniques^7,8^. Direct methods are classified as Open-Circuit Voltage (OCV), Short Circuit Current (SCC), and Hill Climbing (HC). Indirect methods such as Perturb and Observe (P&O) and Increment Conductance (IC)^8^. Some of the modern methods are ANN, Genetic algorithms (GA), and fuzzy logic.

Surveyed previous proposals for classical methods to extract MPPT are presented. OCV and SCC methods have been analyzed in^6^. They are simple methods that need only one current or voltage sensor but have the same disadvantage of low accuracy. For the indirect method, in^8^, the perturbation method has been presented. The disadvantages of this method are that it cannot reach MPP with high precision and oscillates near MPP, making steady state challenging^9,10^.

In^11^, the IC method is presented. It depends on the power curve slope of the PV. However, the disadvantages are the same for P&O. In^12^modified P&O is analyzed. In^12,13^, a modified IC is studied. The modification depends on the variation of step size. In^14^, the study presents valuable insights into hill-climbing MPPT algorithms under low irradiance; however, several limitations exist. The experiments were conducted on a small-scale prototype, additionally, the focus on thin-film PV modules limits the generalization of results to other PV technologies, while environmental factors such as temperature fluctuations not considered. ADC resolutions on algorithm performance was not extensively tested. In^15^, the study highlights several limitations of hill-climbing MPPT algorithms under rapidly changing environmental conditions. The modified algorithms fail to track the true maximum power point under low irradiance, while conventional algorithms such as P&O and INC, although providing satisfactory dynamic response, suffer from significant steady-state power losses. The performance of these algorithms is particularly sensitive to sudden changes in irradiance and temperature, which are common in practical applications like rooftop, building-mounted, or vehicle-mounted PV panels. Moreover, the effectiveness of variable step-size algorithms (P&O, INC) relies heavily on the proper selection of step size, making their performance sensitive to environmental fluctuations. In^16^, the modified P&O algorithm is sensitive to the step size, which affects its performance under significant changes in irradiance or temperature.

In contrast to traditional MPPT approaches, improved performance of PV systems to track the highest power is achieved using artificial intelligence-based MPPT approaches such as ANN, particularly in quickly changing environmental circumstances. In^17^, two hidden layers of ANN with a back-propagation network were used. The input to ANN is only Irradiance, and the output is only PV voltage V_PV_. Using nonelectrical components, G, results in high costs. In^18^, the inputs to ANN are T and G while the output is PPV. This system is more accurate because it uses G and T as inputs that have more effect on the maximum power point of PV. However, this system suffers high costs in addition to using voltage and current sensors in output and indirect control for duty cycle values. In^19^, the ANN has been achieved by direct control. Measurements of current and voltage weren’t needed for comparative output. This reduced cost and was simple to deploy. However, because ANN was not properly trained, a significant error in output power was discovered. In^20^, T, G, and V_OC_ are employed as inputs to ANN while the output is V_PV_. Achieved high performance technique is obtained, and insertion of V_OC_ enhances the tracking. However, this system is expensive, needs extra two voltage sensors, and uses indirect control for duty cycle values.

Since solar plants in Egypt are generally large-scale, the proposed method was designed from a large-scale perspective. By measuring Voc and Isc from only one module—rather than the entire PV array—the sensor ratings and overall cost of proposed method are significantly reduced.”

The objectives and main contributions of this work are to:

- Using a first stage of ANN to estimate solar irradiance (G) and module temperature (T) reduced costs by eliminating the need for a pyranometer, which normally requires additional sensors and a controller for irradiance control, as well as a separate temperature sensor.
- Comprehensive simulation study and comparison of the obtained results using proposed ANN with respect to use conventional methods such as Newton-Raphson (NR) and open circuit voltage (VOC).
- The second stage of the proposed ANN is used to track the Maximum Power Point (MPP) under variations of T, G, and load.
- The variable D calculation block is used to compute different D values at different loads, simplifying the implementation of the proposed ANN.
- The proposed method was simulated under different levels of T, G, and load.
- The proposed method was simulated for a case study in Hurghada.
- Experimental validation of the proposed ANN on PV system in two cases. The first case is implemented inside the laboratory using PV emulators, while the second case is carried out outside the laboratory using three 100 W PV panels.

The paper is organized as follows: “System description” presents the system description. The process of T and G estimation and the tracking stage are investigated in “Methodology”. In “Simulation results and discussion”, two cases of T and G estimation and MPP determination are simulated using MATLAB neural network toolbox. In “Experimental results and discussion”, the proposed technique in this study is verified in two cases experimentally.

## System description

**Fig. 1:**
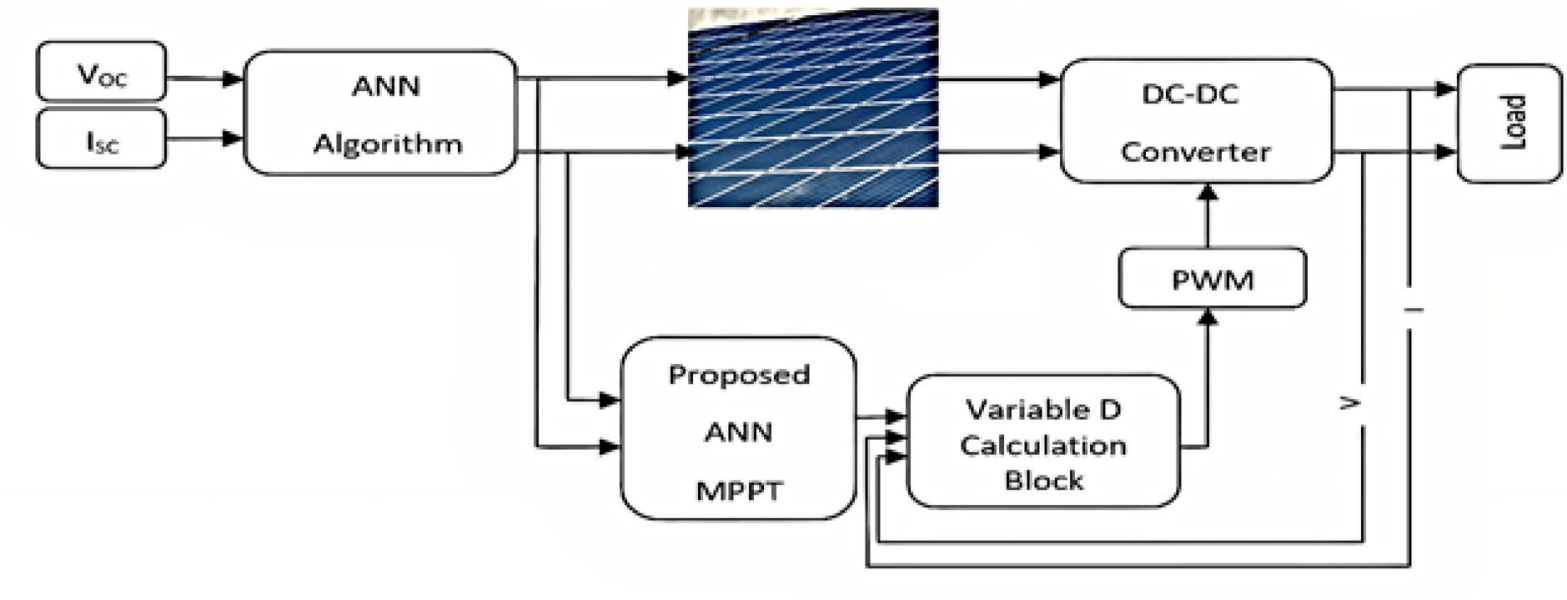
The proposed system block diagram.

The block diagram for the proposed system is shown in Fig. 1. The system consists of a PV panel, DC–DC boost converter, DC load, and two estimation stages. In the first stage, $\:{V}_{OC}$ and $\:{I}_{SC}$ are measured to determine the corresponding T and G values using the ANN algorithm. The optimum power, using ANN MPPT, is then extracted in the second stage by estimating the converter duty cycle corresponding to the obtained T and G values in the previous stage. Moreover, to track load change, the load voltage and current are measured and considered in converter duty cycle determination.

**Fig. 2:**
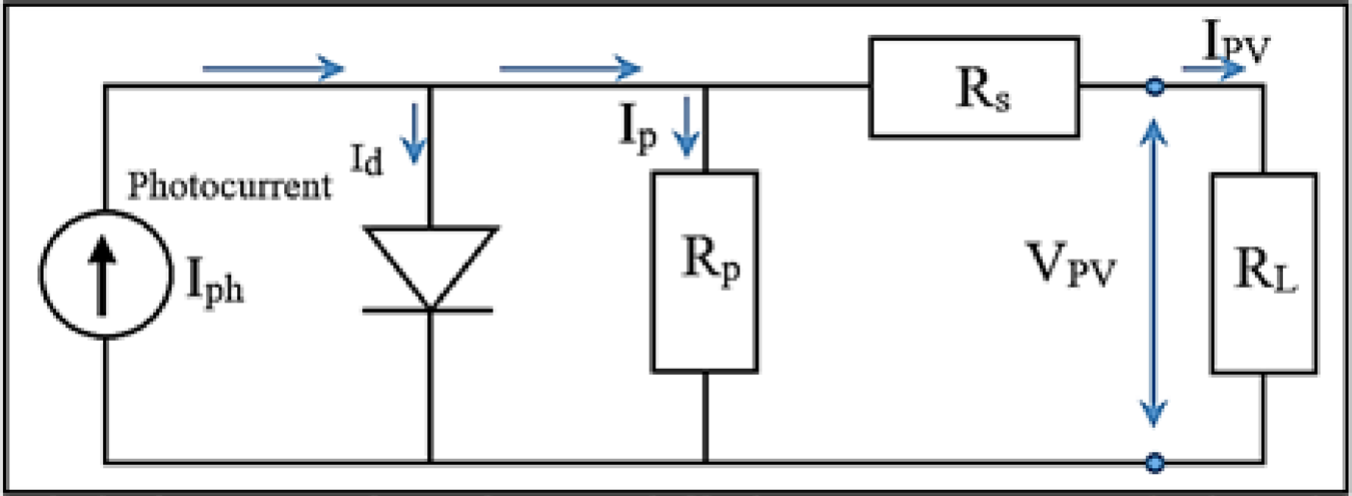
The standard model of the PV module.

The mathematical representation of the PV system using a single-diode model is introduced. The electrical equivalent circuit is shown in in Fig. 2, where $\:{R}_{p}$ is shunt resistance, and $\:{R}_{s}$ is series resistance^21^. Kirchoff’s current rule specifies that the anti-parallel branch to $\:{I}_{ph}$ is substituted by an $\:{I}_{d}$ as in Eq. (1)^22^:

1 $${I_{pv}}={I_{ph}} - {I_d} - {I_p}$$

Where $\:{I}_{ph}$ is the photocurrent, which is generated when a cell is exposed to sunlight. The current traveling through the diode that creates the non-linear features of the solar cell is called $\:{I}_{d}.{I}_{p}$ is the shunt current. The output current is obtained by substituting for $\:{I}_{d}$ and $\:{I}_{p}$ as in Eq. (2)^23^:

2 $${I_{pv}}={I_{ph}} - {I_o}\left[ {{\mathrm{exp}}\left( {\frac{{q\left( {{V_{pv}}+{I_{pv}}{R_S}} \right)}}{{nKT}}} \right) - 1} \right] - \frac{{{V_{pv}}+{I_{pv}}{R_S}}}{{{R_P}}}$$

$\:q$: The electric charge $\:\left(q={1.60210}^{-19}C\right)$. $\:k\::\:\mathrm{Boltzmann}\:\mathrm{constant}\:\left(k={1.380650310}^{-23}J/K\right).$ $\:n$ : The ideality factor. $\:T$ : Temperature of a cell $\:\left(K\right)$. $\:{I}_{o}$: Diode Saturation current ($\:A$). $\:{R}_{S},{R}_{p}$ : Series, and shunt resistance$\:\left({\Omega\:}\right)$.

Several solar cells are commonly connected in series to make a PV module. $\:{N}_{S}$ denotes the number of series cells of a single module. $\:{I}_{M}$, is the module output current, is presented in Eq. (3)^22^:

3 $${I_M}~={I_{ph}} - {I_o}\left[ {\exp \left( {\frac{{q\left( {{V_M}+{I_M}{N_S}{R_S}} \right)}}{{nKT{N_S}}}} \right) - 1} \right] - \frac{{{V_M}+{I_M}{N_S}{R_S}}}{{{N_S}{R_P}}}$$

Where $\:{V}_{M}$ is the output voltage of a module. The PV array consists of groups of shunt and series connection of PV modules. The output current of array $\:{I}_{A}$ can be computed using Eq. (4):

4 $$\begin{array}{*{20}{r}} {{I_A}=}&{{N_P}{I_s} - {N_P}{I_o}\left[ {\exp \left( {\frac{{q\left( {{V_A}+{I_A}\frac{{{N_S}}}{{{N_P}}}{R_S}} \right)}}{{nKT{N_S}}}} \right) - 1} \right] - \frac{{{V_M}+{I_M}{N_S}{R_S}}}{{{N_S}{R_P}}}} \end{array}$$

## Methodology

For the considered system configuration given in Fig. 1, the process of T and G estimation is investigated. Then, the optimum value of the converter duty cycle corresponding to PV MPP is obtained as presented in this section.

### Estimation stage

**Fig. 3:**
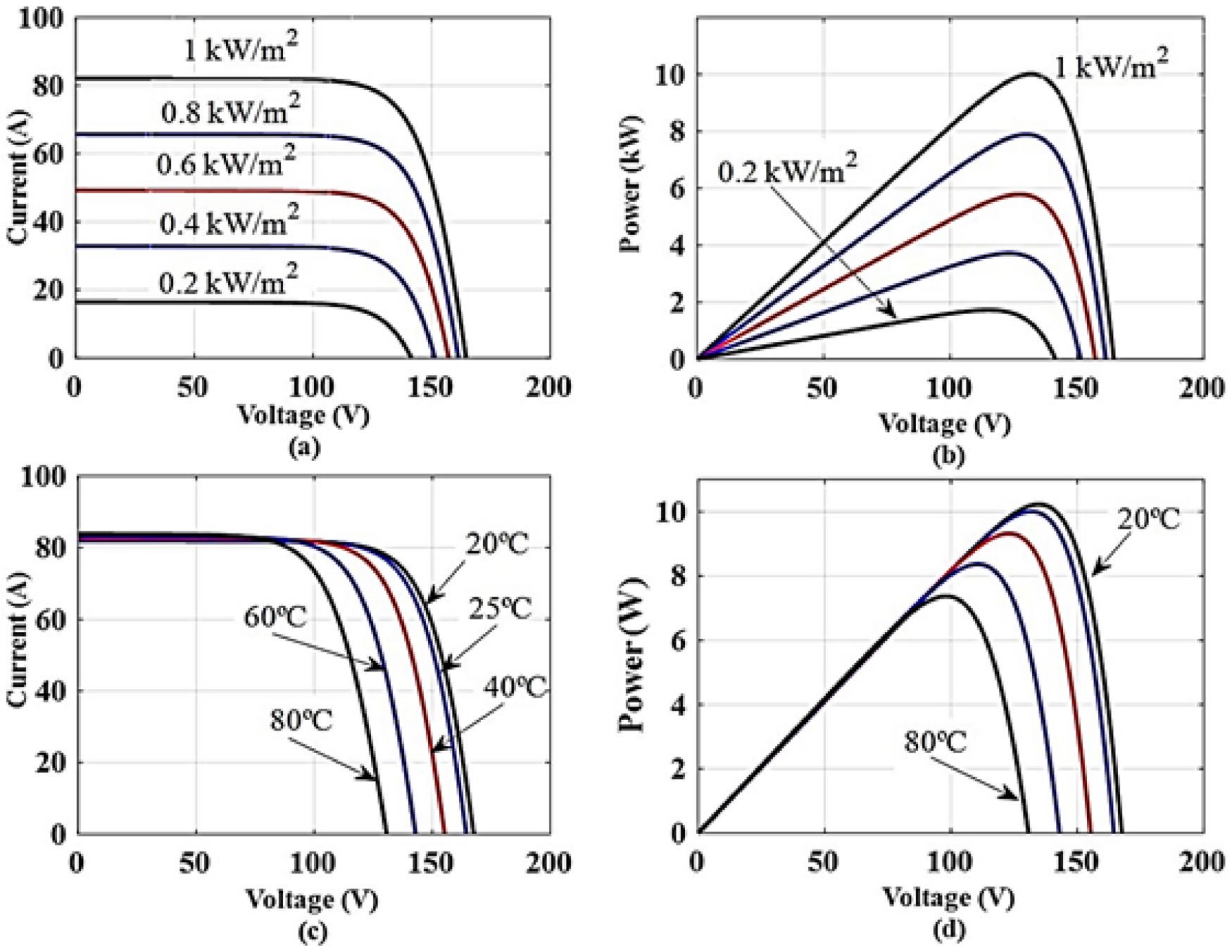
PV array characteristics at various G and T: **a** I–V characteristics at various G, **b** P–V characteristics at various G, **c** I–V characteristics at various T, and **d** P–V characteristics at various T.

The photovoltaic $\:{V}_{OC}$ and $\:{I}_{SC}$ are changed with T and G variation as shown in Fig. 3. In contrast to conventional methods of measuring T and G to determine MPP, in this study T and G are estimated to find MPP directly by only measuring $\:{V}_{OC}$ and $\:{I}_{SC}$ by low-cost sensors.

**Fig. 4:**
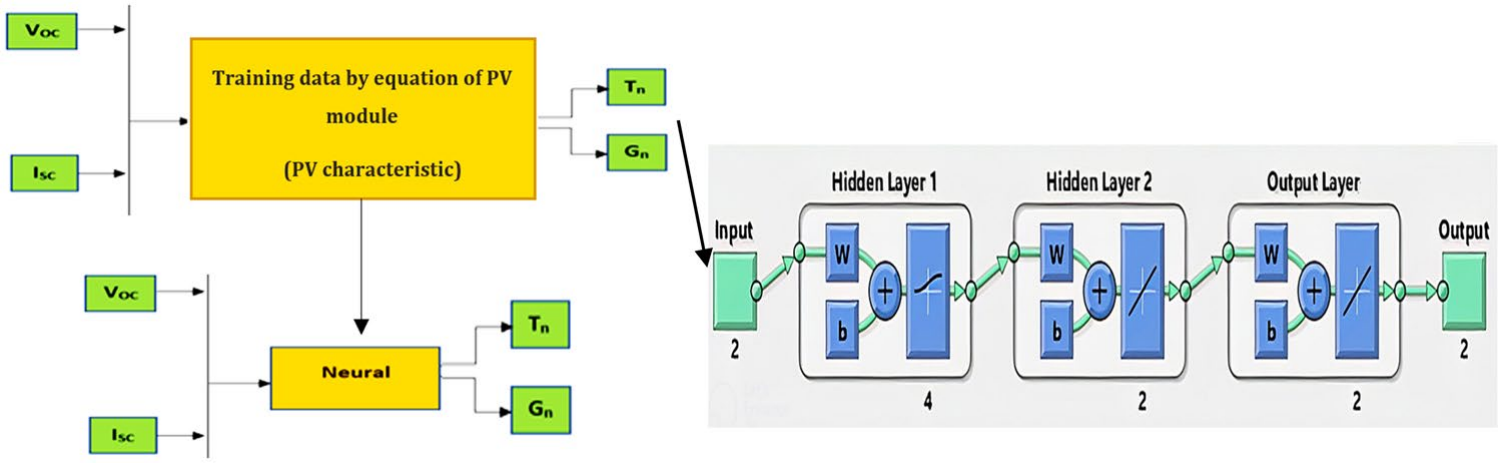
The proposed ANN architecture of estimate T and G.

To achieve this objective, ANN is used in this stage. The ANN receives two inputs and generates two outputs. The two inputs are $\:{V}_{OC}$ and $\:{I}_{SC}$ of the PV module, obtained from the PV characteristics under different temperature and irradiance conditions by using PV module equation. These generated data were then used to train the neural network as shown in Fig. 4. Moreover, the two outputs are the corresponding T and G. To ensure a robust and accurate model, we adopted a backpropagation (BP) approach. This type of ANN is particularly suitable for quantitative studies with smaller datasets as it can handle complex relationships without compromising power or precision.

To assess the model performance, the dataset was divided into training, validation, and test sets, with a recommended split ratio of 70%, 15%, and 15%, respectively. The optimal ANN structure, comprising two hidden layers with eight neurons as in Fig. 4, was determined through iterative adjustments during training to minimize the mean squared error (MSE). After 100 iterations, the best-performing ANN achieved a validation MSE of 0.00089, demonstrating its accuracy in estimating T and G values for the corresponding PV${V}_{OC}$ and ${I}_{SC}$.

### The tracking stage

In this stage, we employed a second ANN, establishing the relationship between $\:T$ and $\:G$ as two inputs and the optimum value of the converter duty cycle corresponding to PV MPP as output. The data set for ANN are calculated using Eq. (5)^19^:

5 $$D=1 - \sqrt {\frac{{{R_{in}}}}{{{R_l}}}}$$

In which $\:{R}_{in}$ denotes the typical input resistance of a solar cell and $\:{R}_{l}$ denotes the resistance of the load. When $\:{V}_{\mathrm{m}\mathrm{a}\mathrm{x}}$ is divided by $\:{I}_{max},{R}_{in}$ is fulfilled. By noting the $\:{V}_{mpp}$ and $\:{I}_{mpp}$ sites in the $\:V-I$ characteristics in various atmospheric circumstances, the various $\:D$ is determined and utilized to train the ANN.

The training function of the backpropagation is Traingdx which uses adaptive learning rate and gradient descent momentum to adjust bias and weight values and speed up learning. Applying the generalized delta rule, accelerate the momentum concept as given in Eq. (6)^24^:

6 $$\nabla {W_{JK}}(P)=\beta \Delta {W_{JK}}(P - 1)+\alpha {y_i}(p){\delta _k}(p)$$

**Fig. 5:**
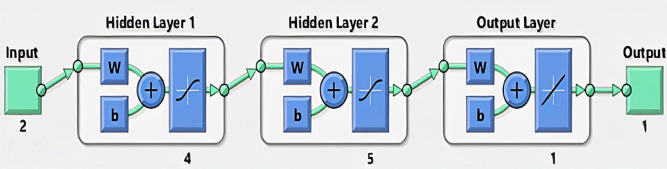
The proposed ANN architecture of track MPP.

When the momentum fixed parameter $\:\left(\beta\:\right)$ is represented by a positive value. Two learning criteria are utilized with adaptive learning rate $\:\left(\alpha\:\right)$. The optimal ANN structure, comprising two hidden layers with ten neurons as in Fig. 5, was determined through iterative adjustments during training to minimize the mean squared error (MSE). After 135 iterations, the best-performing ANN achieved a validation MSE of 0.0075, demonstrating its accuracy in estimating the optimum value of the converter duty cycle corresponding to T and G values.

The actual duty cycle for MPP can really be derived using Eq. (7). For predetermined PV solar cells with constant temperature and irradiance, $\:{R}_{in}$ is constant regardless of the load value. Variations in $\:{V}_{mpp}$ and $\:{I}_{mpp}$ for PV characteristics cause modifications in the value of $\:{R}_{in}$ when T and G are altered. According to the following formula, the new duty cycle for various loads is determined:

7 $${D_{{\mathrm{new~}}}}=1 - \sqrt {\frac{{{R_{{\mathrm{in~}}}}}}{{{R_{{\mathrm{lnew~}}}}}}}$$

The value of the optimal $\:D$ for various loads will be determined by inserting Eq. (7) within Eq. (5) and arriving at Eq. (8).

8 $${D_{{\mathrm{new~}}}}=1 - \frac{{1 - D}}{{\sqrt {\frac{{{R_{{\mathrm{lnew~}}}}}}{{{R_l}}}} }}$$

## Simulation results and discussion

In this section, two cases of T and G estimation are simulated using the MATLAB neural network toolbox to verify the analysis. Moreover, the simulation of two cases of MPP determination is presented.

### Estimation of T and G

The first case of T and G estimation is simulated for data obtained from the PV data sheet, while the second case is simulated for data collected for one day in August in Hurghada City, Egypt.

**Fig. 6:**
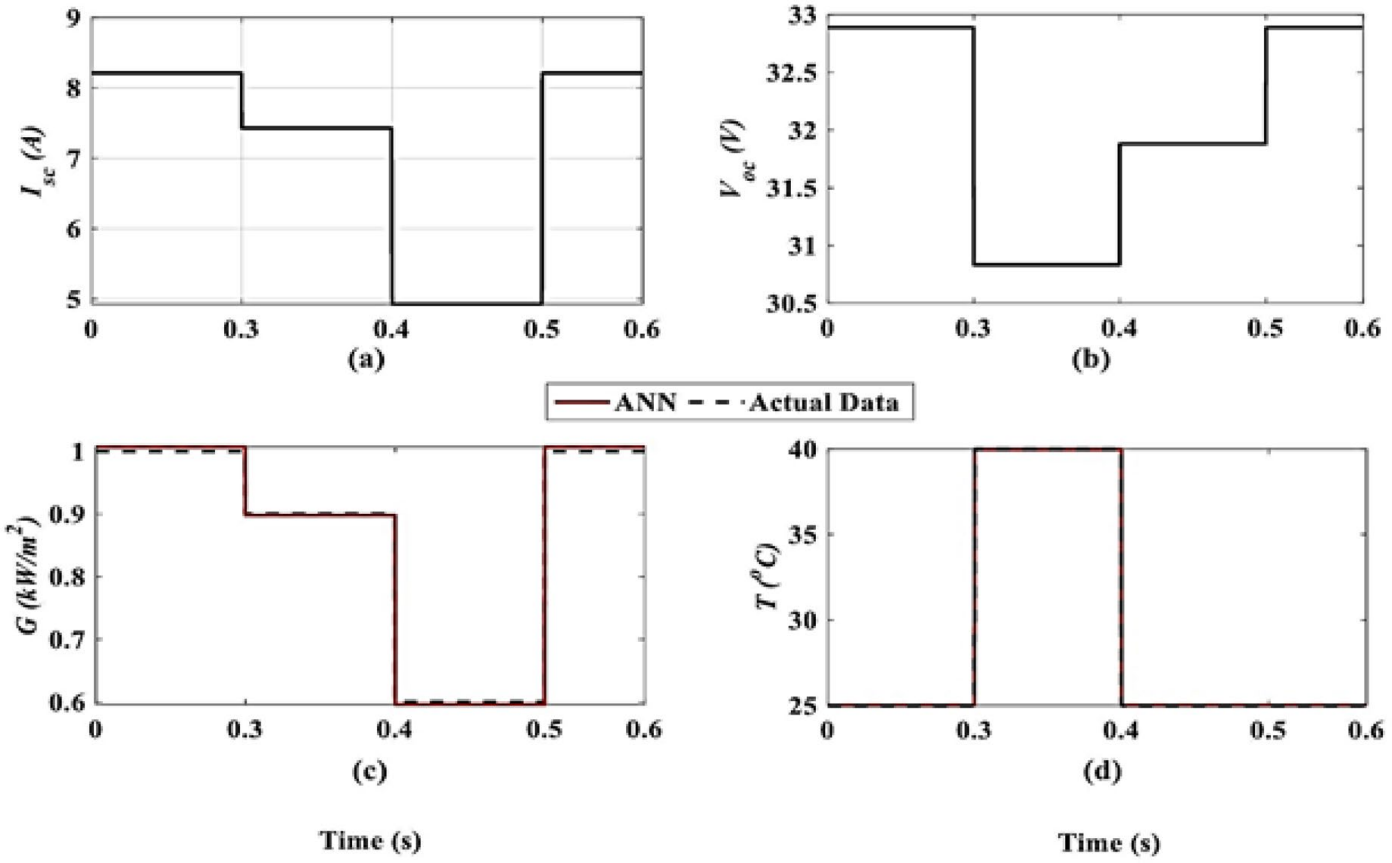
Case I: ANN’s inputs at variance in T and G: **a** $\:{I}_{SC}$ (A), **b** $\:{V}_{OC}\left(\text{}\mathrm{V}\right)$ & ANN’s outputs: **c** $\:\mathrm{G}\left(\mathrm{k}\mathrm{W}/{\mathrm{m}}^{2}\right)$, **d** *T* (°C).

In the first case, T and G are estimated using the proposed ANN at predefined $\:{V}_{OC}$ and $\:{I}_{SC}$ from PV data sheet. The obtained results from ANN are compared to actual data and presented in Fig. 6. The estimated G and T values are compared with Newton-Raphson (NR) and open-circuit voltage (OCV) methods, respectively, and given in Tables 1 and 2. The results from ANN method ($\:{G}_{ANN}$ and $\:{T}_{ANN}$) are so close to actual values, $\:\left({G}_{ACT}\right.$ and $\:\left.{T}_{ACT}\right)$, than NR $\:\left({G}_{NR}\right)$ and OC V ($\:\left.{T}_{OC}\right)$ methods. The maximum error using the proposed ANN for $\:\mathrm{G}\left({E}_{{G}_{ANN}}\right)$ is $\:0.0001\mathrm{\%}$, and the error obtained by the NR $\:\left({E}_{{G}_{NR}}\right)$ method is about 2.5%. The maximum error using proposed ANN for$\:\:\mathrm{T} ({E}_{{T}_{ANN}})$, is 0.24% and $\:\left({E}_{{T}_{OC}}\right)$ is 8.8% by OC method.

**Table 1:** The performance of proposed system for G estimation.

G_ANN_	G_NR_	G_ACT_	${E}_{G_{ANN}}$ %	${E}_{G_{NR}}$ %
1.00011	1.010	1.00	0.0001	0.01
0.900092	0.909	0.90	0.0001	0.01
0.700070	0.708	0.70	0.0001	0.01
0.600061	0.609	0.60	0.0001	0.01
0.500049	0.505	0.50	0.0001	0.01
0.400040	0.39	0.40	0.0001	2.50
0.90	0.91	0.90	0.0001	1.10

**Table 2:** The performance of proposed system for T estimation.

T_ANN_	T_OC_	T_ACT_	${E}_{T_{ANN}}$%	${E}_{T_{OC}}$ %
25.02	25	25.08	0.080	0.32
25.05	25	25.31	0.200	1.24
25.00	25	25.75	0.001	3.00
25.06	25	26.10	0.240	4.40
25.00	25	26.38	0.001	5.50
9.90	10	10.88	1.000	8.80
39.97	40	40.39	0.075	0.98

In the second case, *T* and *G* are estimated using the proposed ANN for real data collected for one day in August in Hurghada City, Egypt. It is located at 27015.5′ N latitude and 33048.7′ E longitude. The data in this study are obtained from NASA surface^25^.

In Table 3, the ANN outputs ($\:{T}_{ANN}$ and $\:{G}_{ANN}$) are given and compared with real data ($\:{T}_{ACT}$ and $\:{G}_{ACT}$). The maximum and average error in $\:T\:\left({{E}_{\:}}_{{T}_{ANN}}\right)$ are 1.9% and 0.87% respectively and in $\:G\:\left({{E}_{\:}}_{{G}_{ANN}}\right)$ are 1.25% and 0.25% in respective order. Thus, the obtained results by ANN are approximately equal to the actual data.

**Table 3:** Comparative study of actual and estimated values for real data case.

Hour	T_ACT_	T_ANN_	${E}_{T_{ANN}}$%	${G}_{ACT}$	${G}_{ANN}$	${E}_{G_{ANN}}$%
7:00	23.9	23.8	0.41	0.40	0.40	0.0001
8:00	23.7	23.8	0.34	0.59	0.59	0.0001
9:00	30.2	29.6	1.80	0.65	0.64	0.2300
10:00	35.6	35.3	0.84	0.86	0.85	0.1100
11:00	36.1	36.0	0.27	0.88	0.88	0.0001
12:00	39.3	39.5	0.76	1.00	1.00	0.0001
13:00	38.4	38.8	1.00	0.96	0.98	1.2500
14:00	34.2	34.3	0.29	0.80	0.80	0.0001
15:00	27.1	26.8	1.10	0.53	0.52	0.1800
16:00	20.2	20.6	1.90	0.26	0.25	0.2500

### MPP determination

**Fig. 7:**
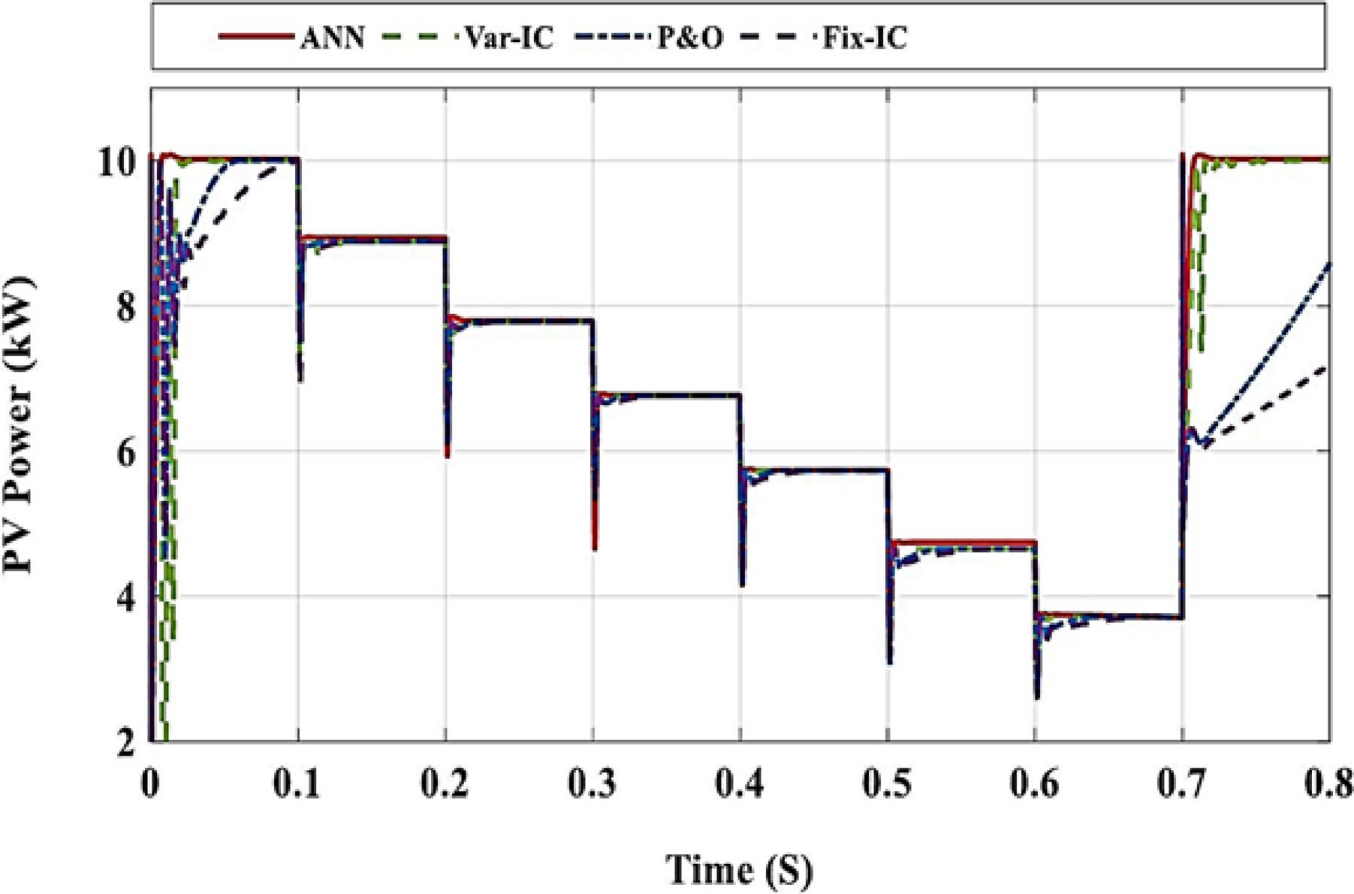
PV output power at constant T and Variance in G.

**Fig. 8:**
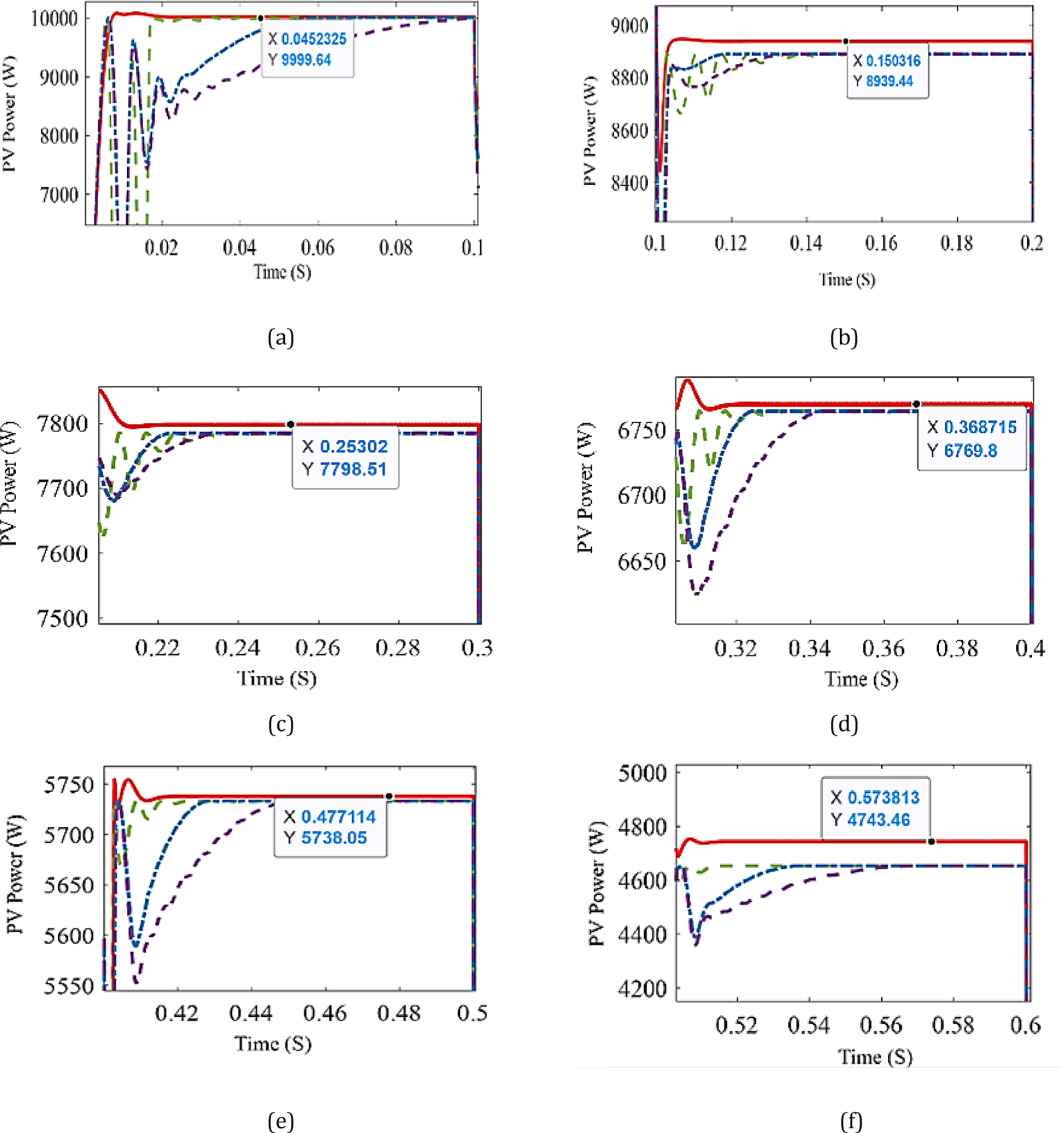

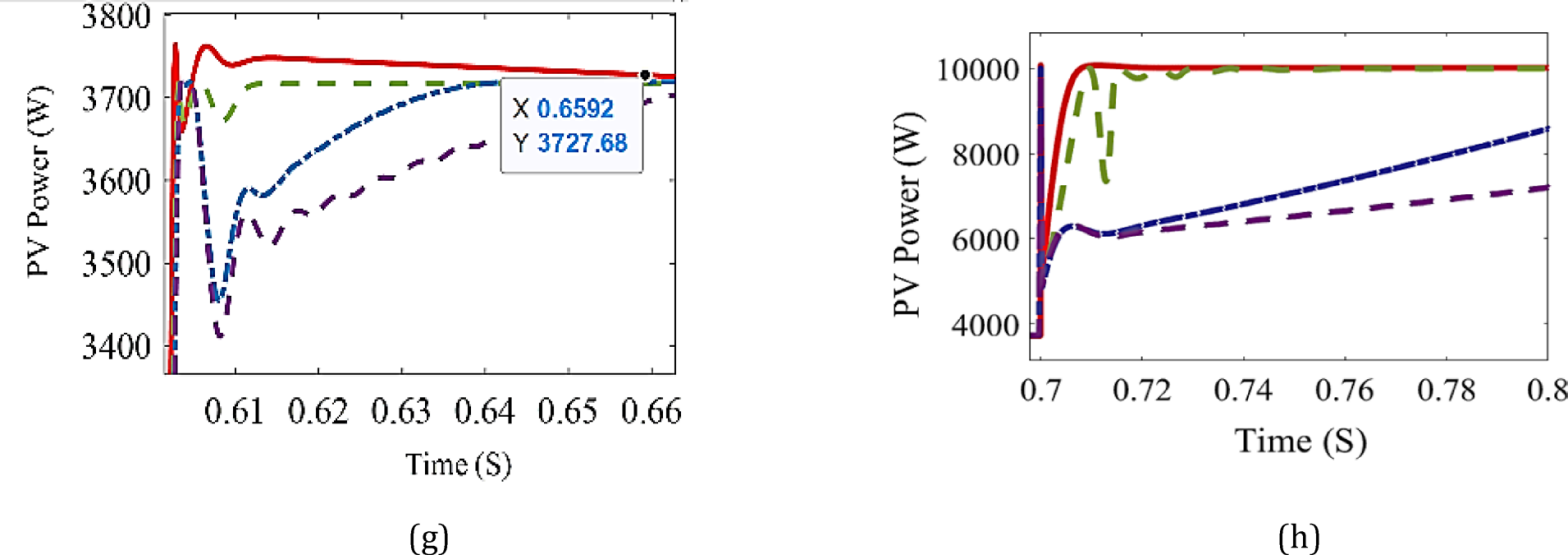
The output power at each stage compared with MPP at T = 25 °C and **a** G = 1000 W/m^2^, **b** G = 900 W/m^2^, **c** G = 800 W/m^2^, **d** G = 700 W/m^2^, **e** G = 600 W/m^2^, **f** G = 500 W/m^2^, **g** G = 400 W/m^2^, **h** G = 1000 W/m^2^.

**Fig. 9:**
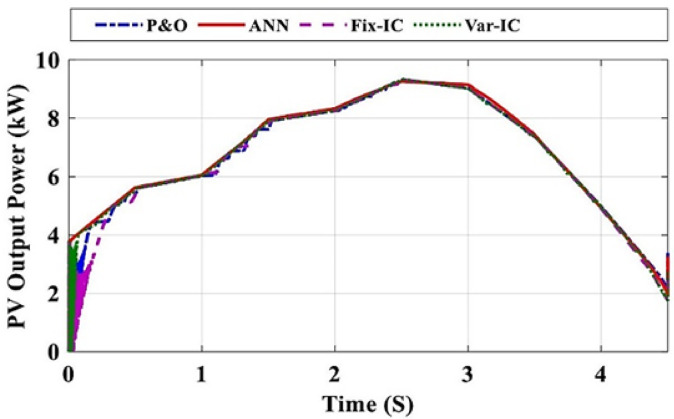
PV output power (real data at Hurghada City).

In this section, the proposed ANN is applied to find the corresponding D for two cases. In the first case, the system is simulated at different radiation levels at a constant temperature of 25 °C. The irradiance level is changed from 1000 W/m² to 400 W/m² with a decreasing step of 100 W/m², and subsequently increased back to 1000 W/m² using the same step size. The output power is given in Fig. 7 while the output power for each radiation level is shown in Fig. 8, and it is clear that the output power obtained using ANN is the closest one to optimal power. The second case is a study of the system for real data obtained from Hurghada City, Egypt. The PV output power using ANN is the best compared to other methods, as shown in Fig. 9. In both cases, the proposed ANN method results are compared to three traditional methods (P&O, FIC,,VIC, FLC, and other techniques) and extract the maximum power. The system’s performance is summarized in Table 4. For the first case, the time capture (TC) of output power is between 0.008 and 0.02, the tracking efficiency (*μ*) is between 99 and 99.99%, the ripple current (∆I_RP_) is between 0.25 and 0.52 and the ripple voltage (∆V_RP_) is between 0.03 and 0.07. Under the EN50530 irradiance test profile, the dynamic efficiency is calculated according to the method presented in^26^. Capture time is defined as the time interval required by the MPPT algorithm to converge and remain within a ± 2% tolerance band around the theoretical maximum power point following a step change in irradiance or load conditions. This metric quantitatively reflects the dynamic tracking capability of the proposed controller. Moreover, steady-state error is defined as the normalized mean deviation between the extracted output power and the theoretical maximum power under steady environmental conditions, computed over a predefined observation window after convergence. This metric is employed to assess the tracking accuracy and stability of the MPPT algorithm in steady-state operation.

**Table 4:** The performance of the proposed system for first Case.

	Stage 1				Stage 2				Stage 3			
	TC	µ	∆I_RP1_	∆V_RP1_	TC	µ	∆I_RP2_	∆V_RP2_	TC	µ	∆I_RP3_	∆V_RP3_
ANN	0.02	99.99%	0.52	0.07	0.01	99.6%	0.49	0.062	0.012	99%	0.47	0.06
FLC^27^	0.06	99.8%	0.9	0.5	0.02	99.2%	0.70	0.3	0.022	98.2%	0.8	0.12
P&O^6^	0.06	99.8%	1.2	0.9	0.02	99%	0.98	0.6	0.025	97.88%	0.82	0.82
FIC^6^	0.1	99.7%	0.6	1.6	0.035	99%	0.72	0.4	0.030	97.87%	0.72	0.68
VIC^13^	0.065	99.8%	1.2	1.1	0.045	99%	1.1	1.28	0.032	97.9%	0.80	0.82
INRES^28^		98.90%		–	–	–	–	–	–	98.76%	–	–
Drift Free P&O^28^		98.65%		–	–	–	–	–	–	98.76%	–	–
Delta P&O^28^		98.40%		–	–	–	–	–	–	98.45%	–	–
Delta INCOND^28^		98.40%		–	–	–	–	–	–	98.45%	–	–
Dual-Tracking^28^		98.9%		–	–	–	–	–	–	98.76%	–	

	Stage 4				Stage 5				Stage 6			
	TC	µ	∆I_PR4_	∆V_PR4_	TC	µ	∆I_PR5_	∆V_PR5_	TC	µ	∆I_PR6_	∆V_PR6_
ANN	0.012	99%	0.37	0.05	0.01	99.7%	0.34	0.042	0.01	99.7%	0.25	0.03
FLC^27^	0.023	98.7%	0.40	0.12	0.02	99.65%	0.40	0.10	0.02	99.2%	0.37	0.09
P&O^6^	0.025	98.5%	0.71	0.85	0.028	99.57%	0.60	0.79	0.035	97.8%	0.46	0.75
FIC^6^	0.04	98.57%	0.56	0.60	0.045	99.85%	0.45	0.44	0.060	97.7%	0.34	0.40
VIC^6^	0.03	98.5	0.65	0.62	0.018	99.85%	0.50	0.50	0.015	97.8%	0.37	0.50
Adaptive step-size^26^		98.67%	–	–	–	–	–	–	–	–	–	–
Voltage- Drift-Free^26^		98.7%	–	–	–	–	–	–	–	–	–	–

## Experimental results and discussion

**Fig. 10:**
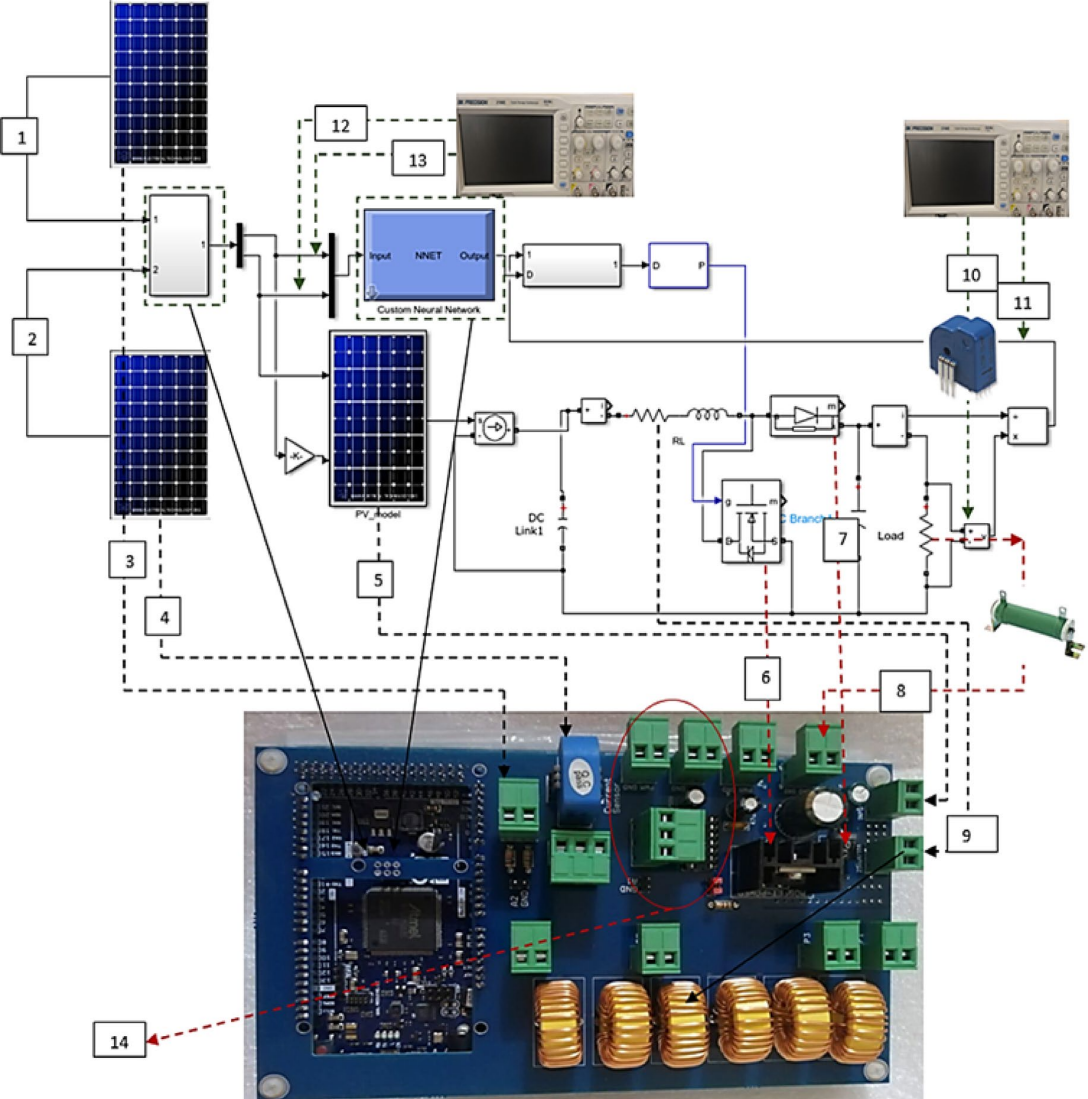
Experimental power and control circuit.

In this section, the proposed technique in this study is verified experimentally in two cases. The experimental power and control circuits are shown in Fig. 10. The first case is implemented inside the laboratory using programmable power supplies as PV emulators, while the second case is carried out outside the laboratory using three 100 W PV panels.

### Case study: indoor experimental setup

**Fig. 11:**
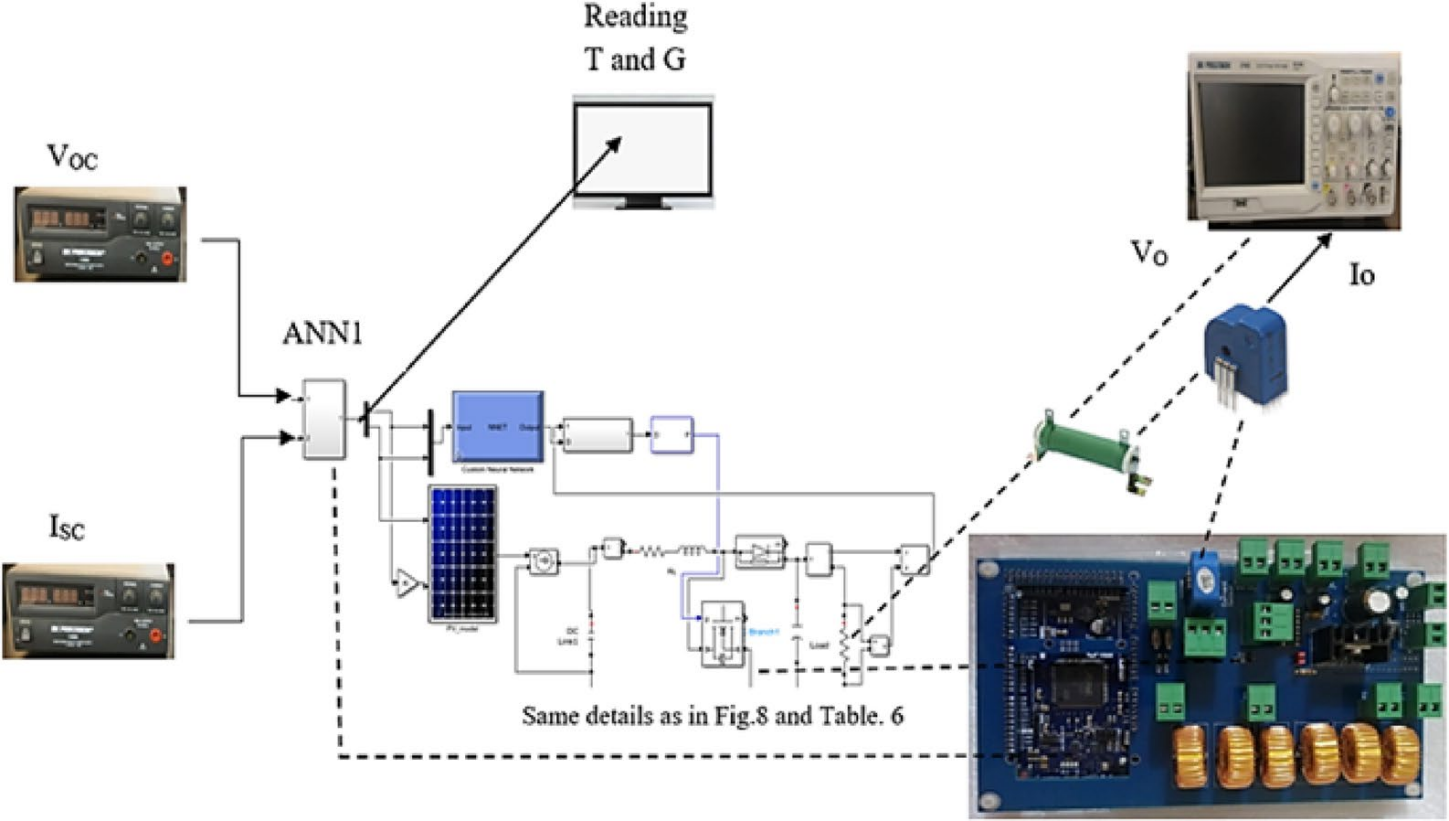
The experimental indoor hardware.

The components used to implement the experimental study here are three power supplies; two of them are power supplies (18–20 A) for open circuit voltage and short circuit current measurement instead of using two PV sources to get different output power values at different temperatures and irradiance. One programmable power supply (30 V–3 A) equivalent to the main PV module, an oscilloscope with two channels, variable power resistance (100 Ω–100 W), and an Arduino duo for the control circuit. The following diagram, shown in Fig. 11, illustrates how the experimental setup was connected in the laboratory.

There are six cases for irradiance and temperature variation are studied and given in.

**Fig. 12:**
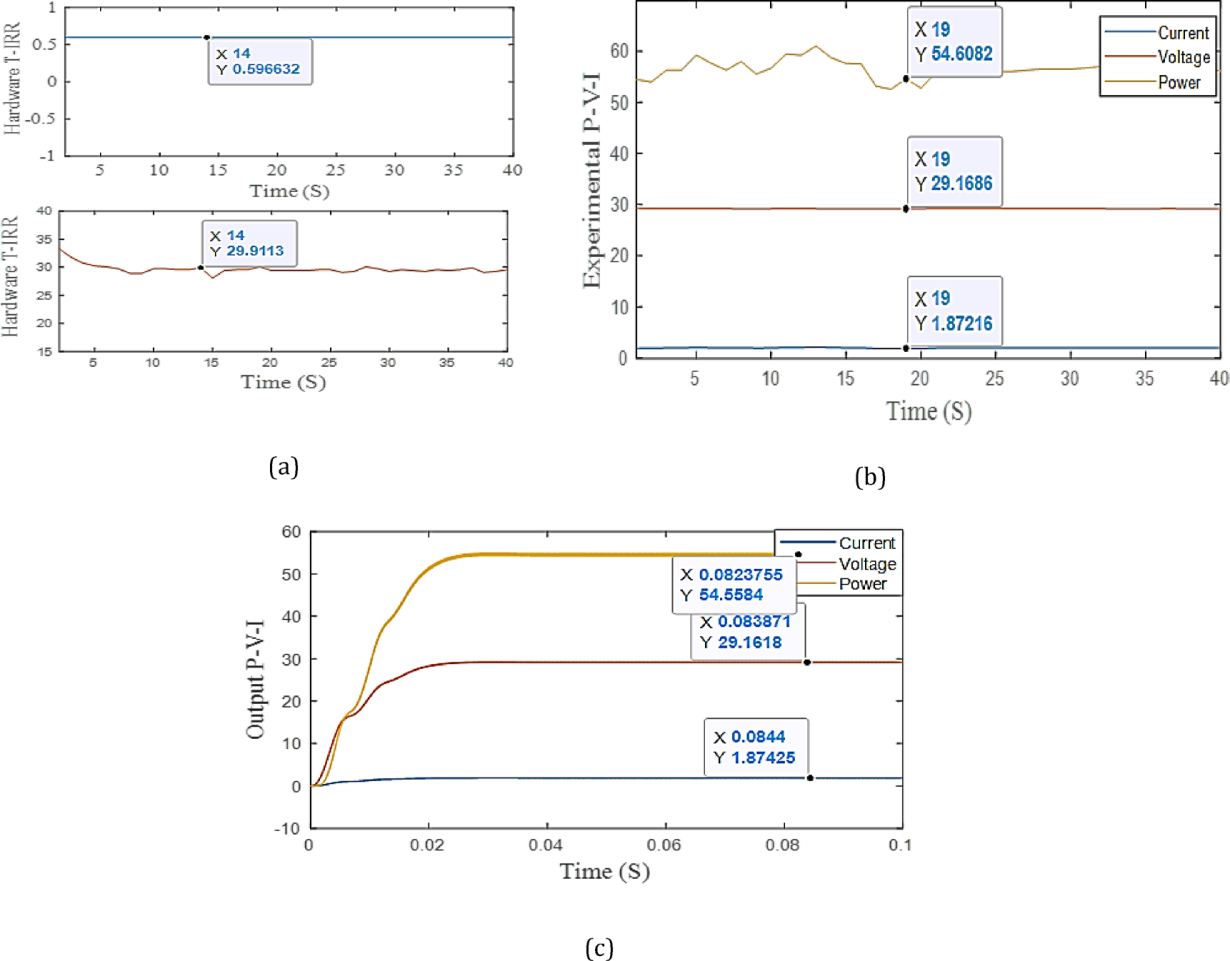
Indoor experimental setup: **a** the output P-V-I of simulation at G = 0.6 kW/m^2^ and T = 30 °C. **b** The experimental output of T and G, **c** the output P-V-I of experimental work.

**Fig. 13:**
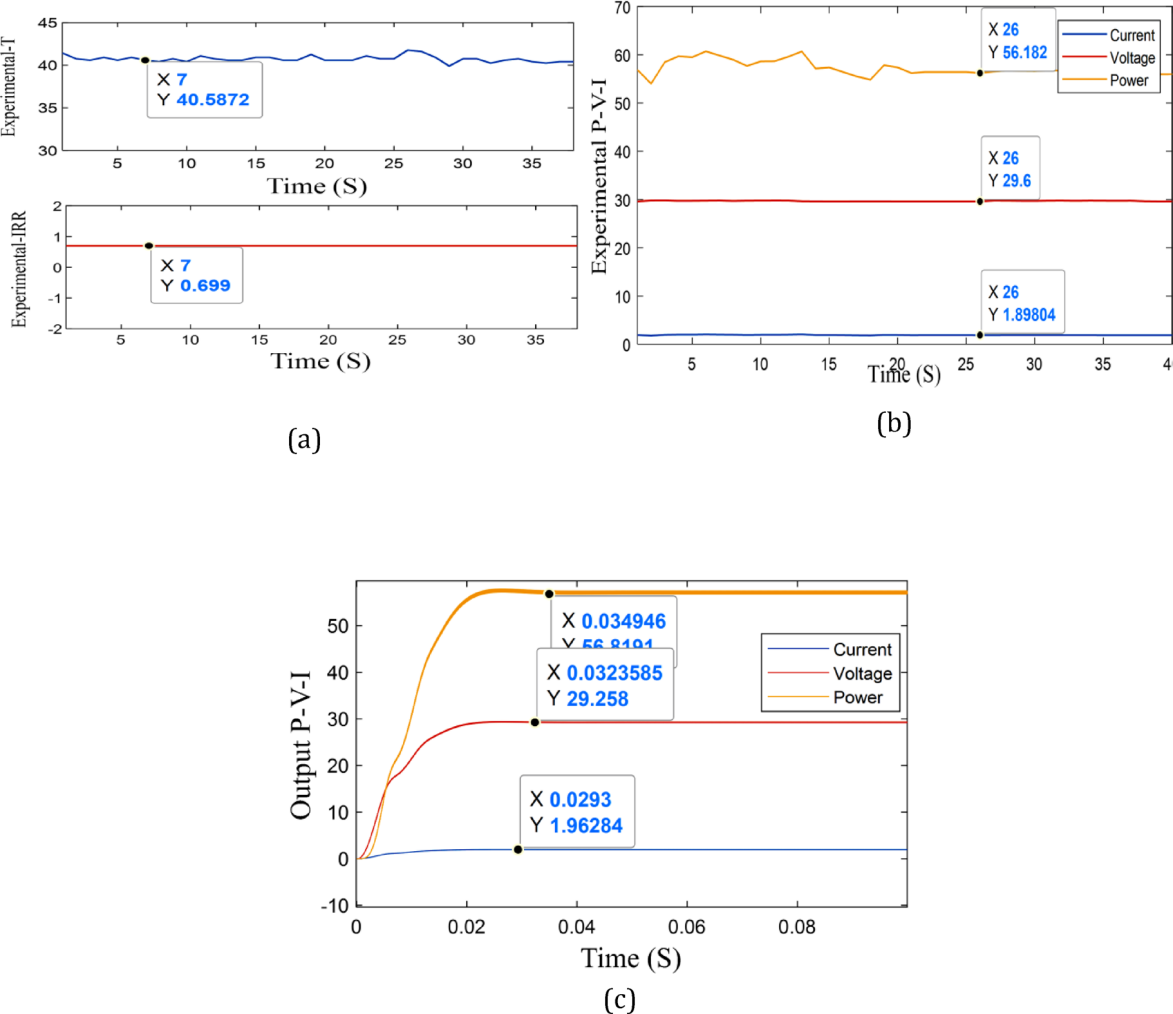
Indoor experimental setup: **a** the output P-V-I of simulation at G = 0.7 kW/m^2^ and T = 40 °C. **b** The experimental output of T and G. **c** the output P-V-I of experimental work.

Table 5. The error for output power is between 0.06 to 0.9%, the error for temperature is between 0.2 to 1.4%, and the error for irradiance is between 0.1 to 1.4%. Two cases of them are shown in Figs. 12 and 13. From the results, the temperature, irradiance, and output power of the experimental system are nearly equal simulation output results.

**Table 5:** The performance results of the system for variable G in kW/m^2 and T in ∘C.

Tact	G = 0.6 & T = 20	G = 0.6 & T = 30	G = 0.7 & T = 25	G = 0.7 & T = 40	G = 0.8 & T = 35	G = 0.8 & T = 45
Tmeas	20.04	29.9	24.7	40.58	35.06	45.57
Terror %	0.2	0.3	1.2	1.4	0.2	1.1
Gact	0.6	0.6	0.7	0.7	0.8	0.8
Gmeas	0.597	0.597	0.69	0.699	0.795	0.798
Gerror %	0.5	0.5	1.4	0.1	0.6	0.2
Vsim	28	29.1	32.39	30.12	31.99	30.39
Vmeas	28.22	29.17	32	30.41	31.63	30.016
Isim	1.804	1.87	2.036	1.896	2.275	2.171
Imeas	1.75	1.872	2.047	1.895	2.315	2.175
Psim	50.48	54.54	65.93	56.82	73.14	65.96
Pmeas	50.51	54.61	65.51	56.18	73.22	65.29
Error P %	0.06	0.1	0.6	0.9	0.1	0.9

### Case study: outdoor experimental setup

In this case, three PV panels (each 100 W) are used^25^. The first PV module is used as a source for$\:{\:V}_{oc}$, the second PV module is used as a source for$\:\:{I}_{sc}$, and the third PV module is used as the main PV module. A current sensor was utilized to measure $\:{I}_{sc}$, whereas a voltage sensor was employed to capture $\:{\:V}_{oc}$. The parameters of the components used in this case are given in Table 6. This study was implemented in October $\:{5}^{th}$, 2022, at EAET academy in EL-salam First, Egypt. The actual values of temperature and irradiance are taken from solar irradiance data (SODA) from 9 AM to $\:2\:\mathrm{P}\mathrm{M}$^29^. The selected location latitude is 30.107 N, and the longitude is 31.565 E.

**Table 6:** The parameters of experimental circuit.

Component no.	Experimental parameter
1	100 W PV module to measure open circuit voltage
2	100 W PV module to measure short circuit current
3	Voltage sensor to measure open circuit voltage (±1%)
4	Current sensor to measure short circuit current (±0.2%)
5	Main 100 W PV module
6	Power MOSFET IRF450N
7	Power diode FR607
8	Variable power resistor 100 Ω (Load)
9	Power coils 47 μH
10	Measurement of load current by oscilloscope
11	Measurement of load voltage by oscilloscope
12	Measurement of irradiance by oscilloscope
13	Measurement of temperature by oscilloscope
14	Driver circuit for power MOSFET: IC IR2112, input for driver 5 V, 12 V, and PWM terminal

**Fig. 14:**
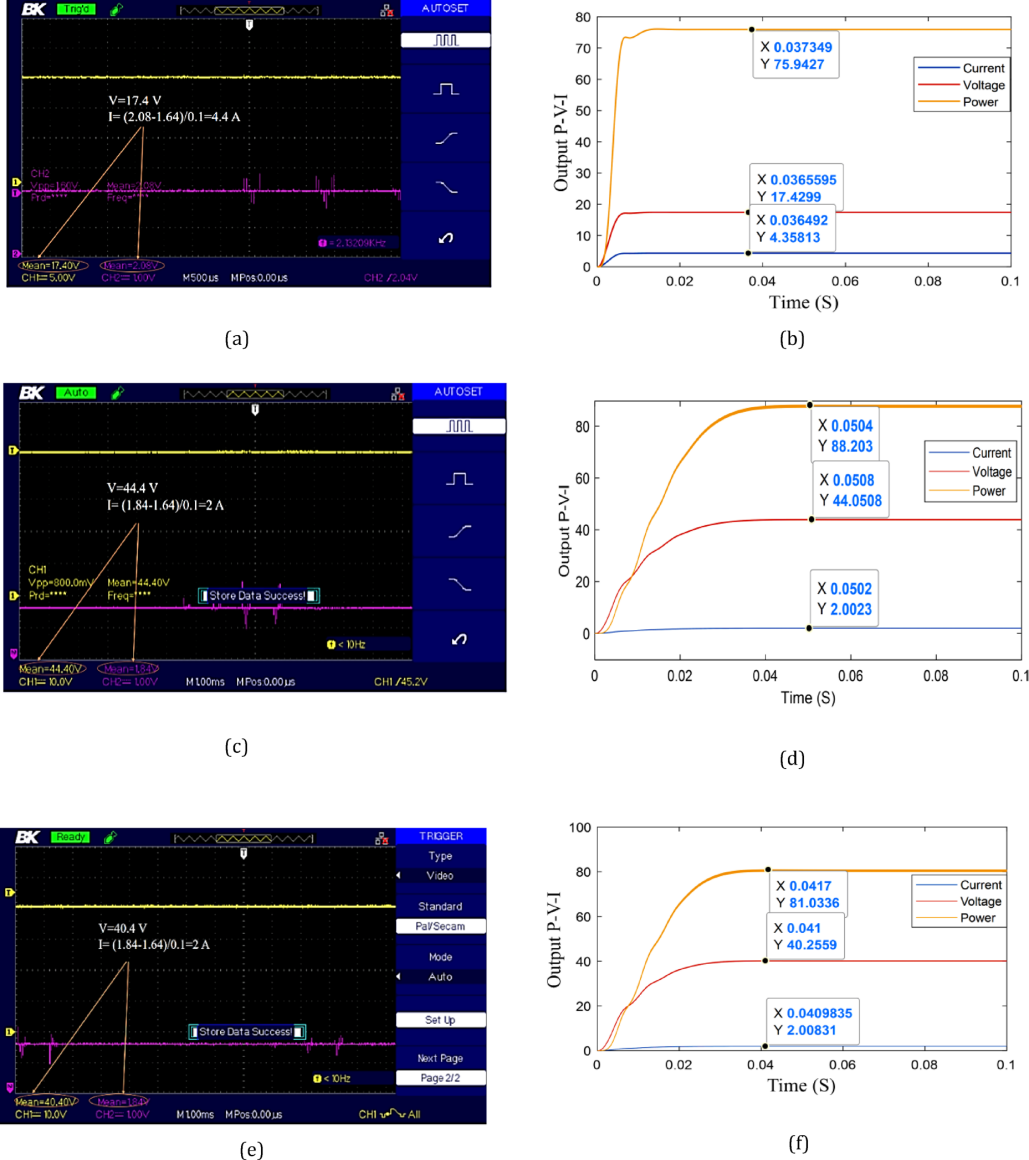
Experimental and simulation results: **a** Experimental data at 9:00:00, **b** Simulation data at 9:00:00. **c** Experimental data at 10:00:00. **d** Simulation data at 10:00:00. **e** Experimental data at 11:00:00. **f** Simulation data at 11:00:00.

**Fig. 15:**
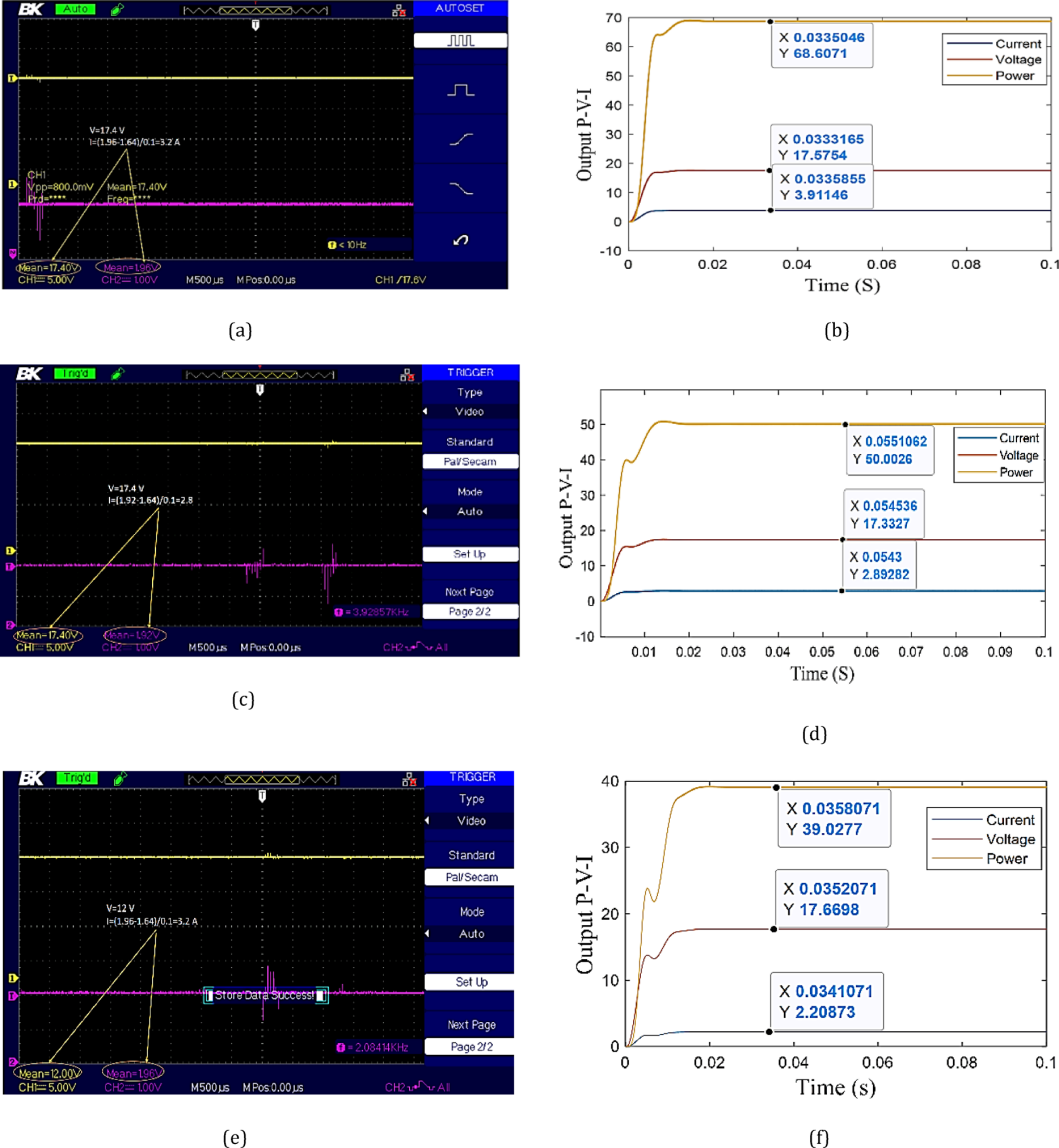
Experimental and simulation results: **a** experimental data at 12:00:00. **b** Simulation data at 12:00:00. **c** Experimental data at 13:00:00. **d** Simulation data at 13:00:00. **e** Experimental data at 14:00:00. **f** Simulation data at 14:00:00.

Irradiation values from HelioClim-3, meteorological data, and temperature are given in Table 7. The experimental output voltage, current, and power are presented in Figs. 14 and 15. The output of current is taken by a current sensor. So, the output must be scaled by the values 1.64 and 0.1 as given in Eq. (9). Where $\:{V}_{\mathrm{sensor\:}}$ is the voltage measurement of the current sensor. The number 1.64 is the reference or zero value of the current sensor and 0.1 is the accuracy of the sensor.

9 $${{\boldsymbol{I}}_{{\boldsymbol{p}}{\boldsymbol{v}}}}=\frac{{{{\boldsymbol{V}}_{{\mathrm{sensor~}}}} - 1.64}}{{0.1}}$$

**Fig. 16:**
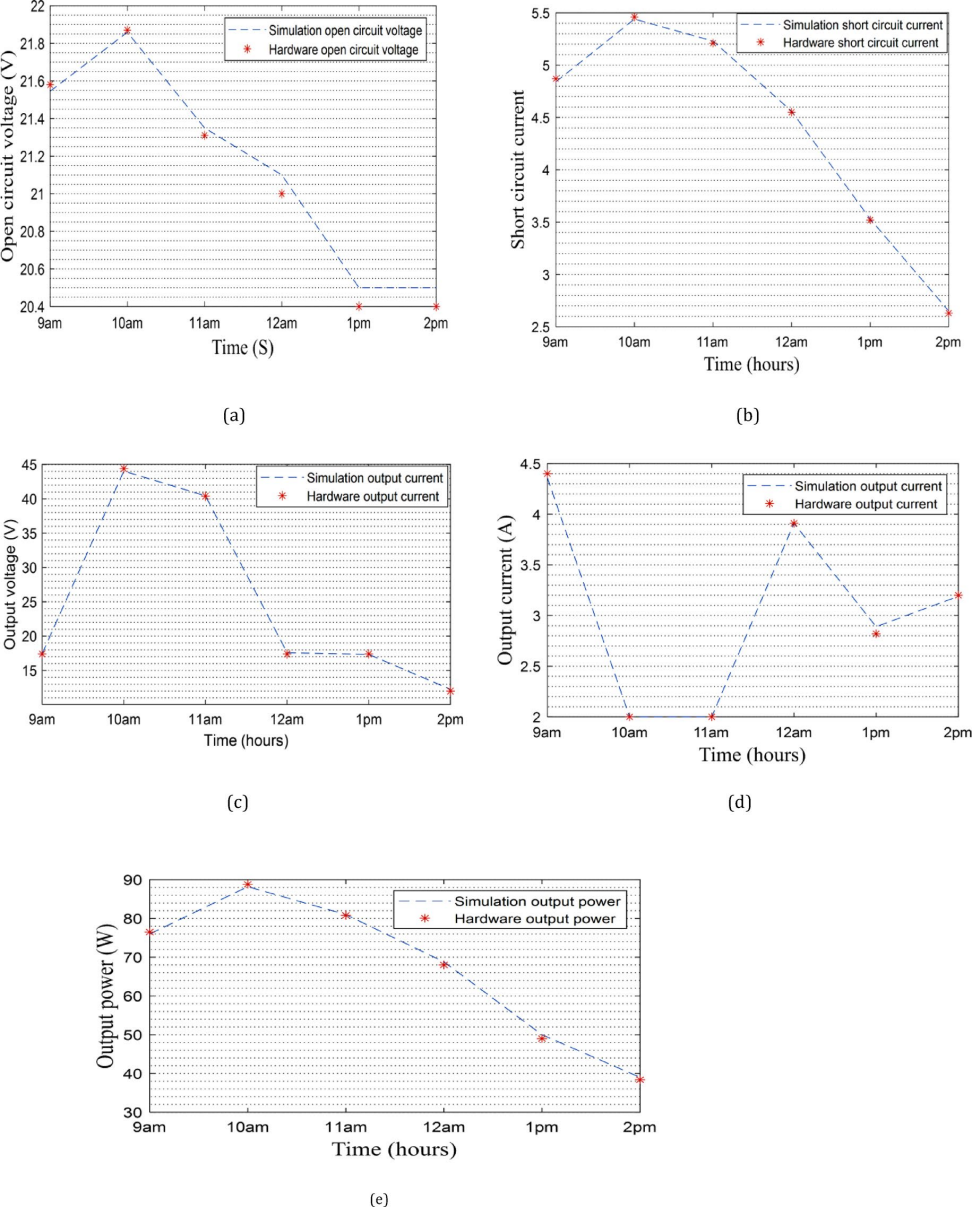
Experimental and simulation results: **a** the simulation and the experimental values of $\:{V}_{oc}$. **b** The simulation and the experimental values of $\:{I}_{sc}$. **c** The simulation and the experimental values of $\:{V}_{o}$. **d** The simulation and the experimental values of $\:{I}_{o}$. **e** The simulation and the experimental values of $\:{P}_{o}$.

The error in the experimental output power is observed at different times of the day as presented in Figs. 14, 15 and 16. From 9:00 AM to 11 AM, the error is between 0.2% and 1%. A significant deviation is recorded at 12:00 PM, where the error reaches 18%. This sharp increase is likely due to the elevated ambient temperature around noon, which negatively impacts the efficiency of solar panels, as their performance generally declines with rising temperatures. After this peak, the error decreases to 2.5% at 1:00 PM and 1.5% at 2:00 PM. These results proved the success of the proposed strategy to estimate the irradiance and temperature as well as extracting MPP.

**Table 7:** Irradiation values from HelioClim-3 and meteorological data on October 5th, 2022.

Data time	Global horizontal	Clear-sky	Atmosphere	Temperature
9:00	1060	822	753	27.25
10:00	1115	871	790	28.41
11:00	1089	847	759	29.57
12:00	985	753	656	30.74
13:00	810	596	500	30.37
14:00	575	390	328	30.01

## Conclusion

This paper presented an ANN-based MPPT approach for photovoltaic systems operating under varying environmental conditions. The proposed method demonstrated fast convergence, high tracking efficiency, and reduced steady-state oscillations compared with conventional and fuzzy logic–based MPPT techniques. Simulation results confirmed the effectiveness and robustness of the proposed approach under different irradiance and temperature profiles, making it a promising solution for efficient photovoltaic energy extraction. A proposed MPPT system based on two artificial intelligence techniques was employed to maximize the extracted power from photovoltaic systems. The first AI-based estimation block was used to accurately estimate the cell temperature and solar irradiance, providing high accuracy with low computational cost. The estimated temperature and irradiance values were compared with Newton–Raphson and open-circuit voltage methods, showing best performance. The second ANN block was utilized to control the duty cycle under varying temperature and irradiance conditions, while load variations were also considered in the analysis. The proposed estimation and MPPT strategy was validated using both simulation and experimental studies with real data obtained from photovoltaic projects in Hurghada and El-Salam cities in Egypt. Two methods are used to carry out the experimental study. The first study employs PV modules in EL-Salam city under real-world T and G values. The second experimental investigation uses DC supplies as the system’s power source rather than PV panels. The results for estimates T and G are much closer to real values than NR and VOC methods. The output power is highest, with lower oscillation and fast response of current and voltage compared with VIC, FIC, and P&O. For the simulation cases, the error for T is 0.001% by the proposed ANN. The error for G is 0.0001% by the proposed ANN. The Time capture is 0.007, the efficiency is 99.99%, the ripple in voltage is 0.018, and the ripple in current is 0.12. For real cases, the temperature or irradiance error is 0.02%. For the first experimental study, the error for output power for most results is 0.01 to 1.1%. The error for temperature is 0.2 to 1.7%, and for irradiance is 0.2 to 1.4%. For the Second experimental study, the error in output power is very small. The error is between 0.2 and 2.5% except at 12 PM.

## Future work

- The effect of partial shading on the PV system’s output power was not considered in this study, but it will be included in subsequent work.
- Machine learning can be used to enhance duty cycle accuracy, and hybrid systems may be explored instead of neural networks.
- The system performance and accuracy could be further enhanced by employing machine learning or deep learning models trained on a larger and more diverse dataset, allowing for better generalization under various environmental and operating conditions.
- The current study does not include experimentally comparison with state-of-the-art methods; however, this will be addressed in subsequent work, including additional experimental validation, to further evaluate and benchmark the proposed approach.
- integration of advanced optimization techniques, such as hybrid or metaheuristic algorithms, to enhance the performance of the proposed MPPT.
- The integration of the proposed ANN-based MPPT system with grid-connected PV installations.
- Improved DC–DC converter topologies to enhance MPPT efficiency and dynamic performance under varying operating conditions.
- Hybrid energy system incorporating a fuel cell will be explored to improve power reliability and overall system performance.

## Data Availability

All data generated or analyzed during this study are included in this published article.

## Abbreviations

*D*: Duty cycle
*D*_*new*_: New duty cycle
${\boldsymbol{E}}_{{{\boldsymbol{G}}_{{{\boldsymbol{ANN}}}} }}$: Irradiance estimation error using ANN
${\boldsymbol{E}}_{{{\boldsymbol{G}}_{{{\boldsymbol{NR}}}} }}$: Irradiation estimation error using NR
${\boldsymbol{E}}_{{{\boldsymbol{T}}_{{{\boldsymbol{ANN}}}} }}$: Temperature estimation error using ANN
${\boldsymbol{E}}_{{{\boldsymbol{T}}_{{{\boldsymbol{OC}}}} }}$: Temperature estimation error using VOC method
G: Irradiance of a cell (W/m^2^)
$${\boldsymbol{G}}_{{{\boldsymbol{ACT}}}}$$: Actual radiation (W/m^2^)
${\boldsymbol{G}}_{{{\boldsymbol{ANN}}}}$: Estimated irradiance using ANN (W/m^2^)
$${\boldsymbol{G}}_{{{\mathbf{mes}}}}$$: Measured radiation (W/m^2^)
${\boldsymbol{G}}_{{{\boldsymbol{NR}}}}$: Estimated irradiance using NR (W/m^2^)
${\boldsymbol{I}}_{{\boldsymbol{A}}}$: Output current of array (A)
${\boldsymbol{I}}_{{{\boldsymbol{max}}}}$: Maximum PV current (A)
$${\boldsymbol{I}}_{{{\mathbf{mes}}}}$$: Measured current (A)
${\boldsymbol{I}}_{{\boldsymbol{o}}}$: Diode Saturation current (A)
${\boldsymbol{I}}_{{{\boldsymbol{SC}}}}$: Short circuit current of PV (A)
$${\boldsymbol{I}}_{{{\mathbf{sim}}}}$$: Simulated current (A)
*k*: Boltzmann constant (k = 1.380650310^−23^ J/K)
*n*: Ideality factor
${\boldsymbol{N}}_{{\boldsymbol{S}}}$: Number of series cells of a single module
$${\boldsymbol{P}}_{{{\mathbf{sim}}}}$$: Simulated power (W)
ANN: Artificial neural network
FIC: Fixed increment conductance
FLC: Fuzzy logic control
HC: Hill Climbing
MPPT: Maximum Power Point Tracking
MSE: Mean Squared Error
*q*: Electric charge (q = 1.60210^−19^ C)
*R*_*in*_: Typical input resistance of a solar cell (Ω)
*R*_*l*_: Load resistance (Ω)
$R_{S} ,R_{p}$: Series, and shunt resistance (Ω)
T: Temperature of a cell (K)
*T*_*ACT*_: Actual temperature (K)
*T*_*ANN*_: Estimated temperature using ANN (K)
*T*_mes_: Measured temperature (K)
*T*_*OC*_: Estimated temperature using VOC method (K)
*V*_max_: Maximum PV voltage (V)
*V*_*M*_: Output voltage of a module (V)
*V*_mes_: Measured voltage (V)
*V*_OC_: Open circuit voltage of PV (V)
V_PV_: PV voltage (V)
$$V_{{{\mathrm{sim}}}}$$: Simulated voltage (V)
$\alpha$: Adaptive learning rate
$\beta$: Momentum fixed parameter
$$\mu$$: Tracking efficiency
∆I_RP_: Ripple current
∆V_RP_: Ripple voltage
$${\mathrm{NR}}$$: Newton–Raphson
${\mathrm{OCV}}$: Open-circuit voltage method
P&O: Perturb and observe
SCC: Short-circuit current
TC: Time capture
VIC: Variable incremental conductance
